# Sport Participation in Early and Middle Adolescence: The Interplay Between Self-Perception and Psychobiosocial Experiences in Predicting Burnout Symptoms

**DOI:** 10.3389/fpsyg.2022.855179

**Published:** 2022-06-13

**Authors:** Milena Morano, Claudio Robazza, Montse C. Ruiz, Laura Bortoli

**Affiliations:** ^1^Parisi-De Sanctis Institute, Italian Ministry of Education, University and Research, Foggia, Italy; ^2^School of Medicine and Health Sciences, “G. d’Annunzio” University of Chieti-Pescara, Chieti, Italy; ^3^Behavioral Imaging and Neural Dynamics Center, Department of Medicine and Aging Sciences, “G. d’Annunzio” University of Chieti-Pescara, Chieti, Italy; ^4^Faculty of Sport and Health Sciences, University of Jyväskylä, Jyväskylä, Finland

**Keywords:** physical self-perception, self-esteem, sport competence, social physique anxiety, burnout

## Abstract

Adolescence is characterized by pubertal physical changes, cognitive development, and modified social expectations. Adolescent athletes often enter a more challenging stage of athletic development associated with increased specialization, and become vulnerable to feelings of burnout. It is therefore important to consider intrapersonal psychological factors that can improve sport participation experiences and prevent burnout. Accordingly, the aim of the current study was to examine the interplay between self-perceptions and emotion-related (i.e., psychobiosocial) experiences (e.g., feeling confident, focused, determined, physically charged, and skillful) in predicting burnout symptoms in adolescents. A sample of 12–14-year-olds (*n* = 338, 176 girls and 162 boys; *M*age = 13.42, *SD* = 1.12) and 15–17-year-olds (*n* = 302, 142 girls and 160 boys; *M*age = 15.78, *SD* = 1.17), participating in individual or team sports, were involved in a cross-sectional study to assess positive and negative self-perceptions, functional and dysfunctional psychobiosocial experiences, and burnout symptoms (i.e., emotional and physical exhaustion, reduced sense of accomplishment, sport devaluation). Path analysis results suggest that higher scores on global physical self-perception, self-esteem, and sport competence were associated with lower burnout symptoms, while higher scores on social physique anxiety were associated with higher scores on sport devaluation. Moreover, self-esteem and sport competence were shown to have significant indirect effects on burnout dimensions via functional psychobiosocial experiences. Differences by gender (*p* < 0.001) and by age category (*p* < 0.001) in the variable scores were also found. Compared to girls, boys reported higher scores on competence, functional psychobiosocial experiences, global physical self-perception, self-esteem, emotional and physical exhaustion, and lower scores on social physique anxiety. Compared to 12–14-year-olds, 15–17-year-olds reported lower scores on global physical self-perception and self-esteem, and higher scores on social physique anxiety, reduced sense of accomplishment, and sport devaluation. This study adds to the literature on burnout by considering the role of intrapersonal factors (i.e., global physical self-perception, self-esteem, sport competence, and social anxiety) in predicting burnout symptoms in adolescent athletes, and the mediating effects of psychobiosocial experiences. From an applied perspective, sport coaches should implement strategies to foster positive self-perceptions, promote pleasant psychobiosocial experiences, and prevent burnout.

## Introduction

Adolescent athletes are at risk of chronic stress exposure due to increased sport specialization, training loads, social pressure from responsibilities outside of sport (e.g., school achievement), and physical, emotional, and cognitive changes (Gustafsson et al., 2011; Harter, 2012; Gerber et al., 2019). Prolonged exposure to different sources of stress can lead to burnout symptoms and dropout from sport (Isoard-Gautheur et al., 2016). Individual implicit beliefs that one’s abilities can be developed through practice, and a focus on learning and self-referenced improvement, have been associated with increased enjoyment and motivation, and thus considered protective factors in youth sport (Gardner et al., 2018). Additional psychological factors, such as positive self-perceptions (e.g., self-esteem and perceived competence), are among the intrapersonal variables that could enhance sport participation experiences and prevent burnout (Weiss et al., 2012).

Self-perceptions are generally considered as the way in which people think about themselves. It is widely recognized that sport participation influences psychological and social health of children and adolescents by improving their emotional well-being and self-perceptions and reducing social anxiety (for reviews, see Holt and Neely, 2011; Eime et al., 2013). Adolescence represents a critical phase in the development of self-perceptions, being characterized by pubertal physical changes, cognitive development, and modified social expectations (Harter, 2012). Self-perceptions incorporate not only self-awareness of personal characteristics and reflection on such knowledge, but also social comparison and evaluations from significant others (Somerville, 2013; Guyer et al., 2014). In sport and exercise contexts, self-perceptions are investigated adopting the broad constructs of self-concept and self-esteem with self-concept representing how people describe themselves, while self-esteem relates to the evaluative feelings about themselves (Sabiston et al., 2019).

### Self-Concept, Perceived Competence, and Social Physique Anxiety

Self-concept is considered as a multifaceted and hierarchically organized construct, with perceptions of personal behavior in specific situations at the base of the hierarchy, and a global self-concept at the top (Shavelson et al., 1976; Harter, 1982; Marsh and Shavelson, 1985). Physical self-concept is regarded as a specific domain, which is of particular value in sport and physical activity contexts as it subsumes relevant information about the body and aspects of physical domains, such as strength, endurance, sport ability, or physical appearance (Fox and Corbin, 1989; Marsh and Redmayne, 1994), and includes attitudes, opinions, and cognitions that a person has regarding their own body. It is considered both as a predictor and as an outcome of physical activity (e.g., Marsh et al., 2006). Physical self-concept develops and differentiates with age through experience and intra and interpersonal confrontation processes. Marsh et al. (1994) developed the Physical Self-Description Questionnaire (PSDQ) to assess nine dimensions related to physical self-concept (i.e., strength, body fat, activity, endurance/fitness, sport competence, coordination, health, appearance, and flexibility) and two general higher-order dimensions named global physical self and self-esteem. The PSDQ is considered the most differentiated instrument for the assessment of physical self-concept (Schipke and Freund, 2012).

Together with a general measure of physical self-concept, the assessment of perceived competence in a sport-specific context can provide additional information about individual’s sport experience. A person’s sense of competence in a specific domain is a key construct in most theoretical models of motivation and is considered reflective of past performances that can be predictive of future behaviors (Ryan, 2019). Perception of competence in a domain can lead to positive outcomes in that domain, influencing how a person acts, feels, and adapts to different environments (Marsh et al., 2017). Sport involvement offers young people the opportunity to learn and develop numerous physical skills and abilities, which in turn enhance their physical self-perceptions. Indeed, research findings in children and adolescents have consistently shown organized sports participation to be positively related to physical self-concept (Balaguer et al., 2012; Amado-Alonso et al., 2018; Morano et al., 2020b), self-esteem, and sport competence (Rubeli et al., 2020).

Gender and age differences in physical self-concept and self-esteem have been reported, with boys usually scoring higher than girls (Klomsten et al., 2004; Hagger et al., 2005). Gender differences can be explained by gender stereotypes, gender role expectations, and greater opportunities for male participants to develop their physical skills (Colley et al., 2005; Çağlar, 2009). Regarding age differences, previous studies showed that middle and late adolescents tend to score lower in physical self-perception than early adolescents (Marsh, 1998; Maïano et al., 2004). This may reflect an optimistic self-perception bias in young children and a more realistic and accurate evaluation in older children (Marsh et al., 2015). Beyond the biological factors (i.e., genetic vulnerability, pubertal hormones, pubertal timing and development) that contribute to gender differences (Hyde et al., 2008), boys and girls progressively interiorize gender stereotypes and gender role expectations as they grow older. As a result, physical self-concept becomes more complex and differentiated, but it also tends to decline during adolescence especially in girls (Çağlar, 2009; Arens and Hasselhorn, 2014). For example, Klomsten et al. (2004) found gender and age interactions in both global physical concept and self-esteem, with older girls reporting the lowest ratings.

Global physical self-concept and self-esteem have also been studied in relation to other body related constructs, such as social physique anxiety, a feeling of distress associated with the perceived evaluation of one’s physical self (Frederick and Morrison, 1996), particularly in situations where one’s body is observed or evaluated by others (Hart et al., 1989). Social physique anxiety can play a key role in influencing an individual’s emotional responses and level of effort in exercise and sport settings, given the centrality of the body in these contexts (Mülazımoğlu-Balli et al., 2010). Higher levels of social physique anxiety have been reported in adolescence, especially in girls, and have been linked to low physical self-perceptions and body dissatisfaction, as well as unhealthy eating attitudes and behaviors (Smith, 2004; Alcaraz-Ibáñez et al., 2020).

### Psychobiosocial Experiences

In sport, emotional experiences can be examined by taking a holistic perspective which considers emotions to be central aspects of the so called psychobiosocial experiences (Hanin, 2000, 2007). Psychobiosocial experiences encompass situational (state-like) or relatively stable (trait-like) emotional and non-emotional expressions of subjective feelings that manifest themselves in psychological, biological, and social components related to past, present, and future (anticipated) performances (Hanin and Ekkekakis, 2014). Athletes’ experiences can be pleasant and functional, such as feeling confident and joyful while anticipating future success, or unpleasant and dysfunctional, for example feeling apprehensive about the upcoming competition. These experiences include a main affective component and several non-emotion interactive modalities (i.e., cognitive, motivational, volitional, bodily, motor-behavioral, and operational) that exert functional (i.e., facilitating) or dysfunctional (i.e., debilitating) effects on performance (Ruiz et al., 2017b; Robazza and Ruiz, 2018; Ruiz and Robazza, 2020). Psychobiosocial experiences account for individuals’ perceptions of personal resources to deal with performance and competition demands (see Ruiz et al., 2016). A positive appraisal reflects enough perceived personal resources to deal with task requirements and is expected to lead to functional experiences for performance. On the other hand, a negative appraisal occurs when task demands are perceived as exceeding personal resources and is expected to conduct to dysfunctional experiences for performance.

Psychobiosocial experiences in youth sport have been associated with contextual determinants, such as motivational climate and basic psychological needs satisfaction (Bortoli et al., 2009, 2011, 2012; Ruiz et al., 2017a,^2019^). The results of these studies consistently showed that a perceived mastery (task-involving) climate, in which the coach’s focus is on skill improvement, individual progress, and cooperation with others, was related positively to pleasant and functional experiences, while a performance (ego-involving) climate, in which the coach’s emphasis is on normative-based evaluation, competition, and social comparison between participants, was positively associated with unpleasant and dysfunctional experiences. Psychobiosocial experiences were also found to mediate the effects of basic psychological need satisfaction (i.e., autonomy-choice, competence, and relatedness) on burnout symptoms (Morano et al., 2020a). The results provided evidence of the protective role of functional experiences against burnout symptoms and the detrimental effects of dysfunctional experiences.

### Burnout

Athlete burnout is regarded as a multidimensional syndrome characterized by physical/emotional exhaustion, reduced sense of accomplishment, and sport devaluation (Raedeke and Smith, 2001). It is a dysfunctional condition associated with deleterious affective, cognitive, motivational, and behavioral consequences (Gustafsson et al., 2011, 2018; Gerber et al., 2018a). In a systematic review, Goodger et al. (2007) identified five psychological correlates of athlete burnout, namely, motivation, coping with adversity, response to training and recovery, role of significant others, and identity. Perfectionistic concerns, trait anxiety, ineffective coping skills, high investments, and social constraints are among the vulnerability factors, while social support, perceived autonomy, a supportive motivational climate, and mental toughness are deemed protective factors (Gerber et al., 2018b,^2019^).

Several studies have shown that participation motives and burnout differ by gender, age, and type of sport or exercise (Butcher et al., 2002; Harris and Watson, 2014; Ley, 2020). Burnout tends to develop progressively over time, and as young athletes grow older, they are more likely to experience symptoms of exhaustion and reduced sport accomplishment (Harris and Watson, 2014). In a longitudinal study examining the development of burnout among elite level athletes aged 13–18 years, Isoard-Gautheur et al. (2015) found unique patterns of burnout perceptions. Their findings indicated that reduced sense of accomplishment linearly decreased during adolescence (i.e., growth is accompanied by a higher sense of accomplishment) and was higher for girls than boys, sport devaluation increased over time, and emotional and physical exhaustion remained relatively stable. However, research in adolescent athletes has shown that burnout is an individual experience which develops differently and may vary across sports and situations (Gustafsson, 2007).

Developmental changes in sport related experiences and burnout have often been examined from a psychosocial perspective, particularly in light of motivational theories that emphasize the role of contextual factors, such as achievement goal theory (Nicholls, 1984; Ames, 1992), self-determination theory (Ryan and Deci, 2017), and basic psychological need satisfaction “mini” theory (Ryan and Deci, 2017; Ryan et al., 2021). For instance, mastery (task-involving) climates created by coaches have been found to play a protective effect against burnout (Vitali et al., 2015). Protective effects have also been observed in contexts nurturing psychological needs satisfaction, intrinsic motivation, identified regulation, and integrated regulation (Li et al., 2013; Morano et al., 2020a). Beyond the contextual effects, Smith et al. (2019) have also underlined that personal factors and characteristics can play an important role in influencing individual appraisals and subsequent experiences in sport, which may eventually lead to burnout. Personal factors may entail individual perception of inability to face environmental demands (e.g., balancing sport with school), relatively high social constraints for sport participation from significant others (e.g., parents) combined with low perceptions of control over the sport involvement, motivational characteristics (e.g., high ego-orientation), and personality traits (e.g., perfectionism).

Personal factors are also included in an integrated model of athlete burnout that Gustafsson et al. (2011) proposed to stimulate empirical research and provide sport scientists, coaches, and practitioners with practical indications to prevent maladaptive outcomes. The model incorporates antecedents (e.g., excessive training, stressful social relations), early signs (e.g., mood disturbance, diminished motivation, performance decrement), vulnerability factors (e.g., personality, coping, environmental factors), and consequences (e.g., partial or complete withdrawal from sport). As Gustafsson et al. (2017) noted, there is large body of evidence suggesting that personality factors are associated with athlete burnout, such as coping skills, hope, perceived control, optimism, and perfectionism. Therefore, research on additional adaptive and maladaptive psychological antecedents and possible mediators that could be associated with burnout symptoms is expected to advance the current knowledge base in this area.

### Study Purposes

Adolescent athletes are at high risk of developing burnout symptoms that decrease performance and motivation and can lead to dropout (Gustafsson et al., 2011, 2018; Isoard-Gautheur et al., 2016). For example, in a systematic review and meta-analysis of dropout rates in youth soccer (Møllerløkken et al., 2015) the annual weighted mean dropout rate was 23.9%, with higher rates for girls (26.8%) compared to boys (21.4%). It is therefore crucial to examine the role of intrapersonal psychological factors that can enhance the sport experience and prevent burnout and possible dropout. As stated above, research on adaptive and maladaptive psychological antecedents and possible mediators associated with burnout symptoms should contribute to the current knowledge in this area. According to the integrated model of athlete burnout (Gustafsson et al., 2011), in the present study physical self-perception, self-esteem, and sport competence were posited as adaptive antecedents, while social physique anxiety was assumed as maladaptive. Furthermore, the role of psychobiosocial experiences was also investigated. To date, the only study examining the relationships between burnout symptoms and psychobiosocial experiences in youth sport showed that functional experiences were negatively related with burnout symptoms while dysfunctional experiences were positively related (Morano et al., 2020a). The focus of their study was on environmental factors (i.e., satisfaction of basic psychological needs) and the mediating effect of psychobiosocial experiences on burnout. However, intrapersonal factors were not considered. Therefore, the current study aimed to contribute to the existing literature on burnout in sport by further investigating critical intrapersonal factors which have so far received little research attention. We also aimed to extend the current knowledge from anxiety (e.g., Gomes et al., 2017) or other emotions (e.g., González-García et al., 2020) associated with burnout symptoms to a broader range of athletes’ experiences, including emotional, cognitive, motivational, volitional, bodily, motor-behavioral, and operational feelings, in order to examine further possible variables that can prevent burnout.

In summary, the focus of the current study was on critical self-perceptions in sport deriving from adolescents’ general life experiences during growth and development in different environments (e.g., family, school, social relationships). Specifically, we examined the interplay between self-perceptions (i.e., global physical self-perception, self-esteem, sport competence, social physique anxiety) and psychobiosocial experiences in predicting burnout symptoms in young athletes. We expected: (a) global physical self-perception, self-esteem, and sport competence to be positively related to functional psychobiosocial experiences, and negatively related to dysfunctional psychobiosocial experiences and burnout symptoms; (b) social physique anxiety to be positively linked to dysfunctional psychobiosocial experiences and burnout symptoms, and negatively linked to functional psychobiosocial experiences; (c) functional and dysfunctional psychobiosocial experiences to be associated negatively and positively with burnout symptoms, respectively. We also expected to find significant indirect effects in the relationship between global physical self-perception, self-esteem, sport competence, social physique anxiety and burnout symptoms via functional and dysfunctional psychobiosocial experiences.

A second purpose of this study was to investigate possible age- and gender-related differences in both early and middle adolescents. While middle adolescence has been the target of a great deal of research, early adolescence has been relatively neglected (Blum et al., 2014). According to previous study findings (Morano et al., 2020a), we expected girls and older children to report lower scores on functional psychobiosocial states (or experiences), sport competence perception, and self-esteem, and higher scores on social physique anxiety and burnout compared to boys and the younger cohort, respectively.

## Materials and Methods

### Participants and Procedure

The sample consisted of 642 adolescents aged 12–17 years (318 girls and 322 boys; *M*age = 14.54, *SD* = 1.64), drawn from several sport clubs located in Central Italy and involved in various types of individual sports (e.g., artistic gymnastics, fencing, skating, swimming, tennis, track and field) and team sports (e.g., basketball, futsal, handball, rugby, soccer, volleyball). The request for participation in the study was sent to the sport clubs by the Italian Olympic Committee of the Abruzzo Region. Less than 3% of the contacted participants refused to take part in the study. The participants engaged in a minimum of three 2 h training sessions per week. For the study purposes the sample was divided into two age categories of 12–14-year-olds (*n* = 338, 176 girls and 162 boys; *M*age = 13.42, *SD* = 1.12), pertaining to the early adolescence category, and 15–17-year-olds (*n* = 302, 142 girls and 160 boys; *M*age = 15.78, *SD* = 1.17), pertaining to the middle adolescence category (Haywood and Getchell, 2020).

The study was approved by the ethics committee of the University of Chieti-Pescara (Italy) and was conducted according to the Declaration of Helsinki. Agreement to conduct the study was sought from sport managers and coaches after the purpose of the study was explained to them. All participants’ parents provided written informed consent. Data collection took place before regular training sessions during the competitive season, within training facilities, in secluded locations, and in groups of up to five participants from the same team. Research assistants with experience in data collection administered the questionnaires. Participants were informed that there were no right or wrong responses and assured that their answers would remain strictly confidential.

### Measures

#### Global Physical Self-Perception and Self-Esteem

We used two scales of the PSDQ originally developed by Marsh et al. (1994) and adapted into Italian language (Meleddu et al., 2002). Six items were used to measure global physical self-perception or the extent to which one feels positive about one’s physical self (e.g., “I feel good about the way I look and what I can do physically”), and six items measured self-esteem or the overall positive feelings about oneself (e.g., “Overall, I have a lot to be proud of”). Items are scored on a 6-point Likert scale ranging from 1 (false) to 6 (true). In the Italian version, acceptable internal consistency coefficients of the global physical self-perception and self-esteem scales were reported, with Cronbach’s alpha values of 0.90 and 0.77, respectively (Meleddu et al., 2002).

#### Sport Competence

A single item with an 11-point Likert-type scale ranging from 1 (*very poor*) to 11 (*excellent*) assessed perceived sport competence in relation to body functionality in the participants’ sport-specific context. Specifically, the participants were asked: “What is the level of your physical skills in your sport?” Several authors (e.g., Tenenbaum and Gershgoren, 2011; Bruton et al., 2016; Tenenbaum et al., 2007) have provided a strong rationale for the use of single-item scales which have high face validity and were used in previous research in the sport context (Grossbard et al., 2007). Single-item scales are suggested to be appropriate to assess a given construct as a whole (Nagy, 2002). They are also easy to interpret, ensure adherence of participants, and reduce potential fatigue and boredom associated with item responding (Bruton et al., 2016).

#### Social Physique Anxiety

We used the 7-item version of the Social Physique Anxiety (SPA-7; Motl and Conroy, 2000) to measure anxiety related to perceived negative evaluation of one’s physique by others. The items are scored on a 5-point Likert scale with anchors ranging from 1 (*not at all*) to 5 (*extremely*), including statements such as “In the presence of others, I feel apprehensive about my physique,” or “When I look in the mirror, I feel good about my physique.” Good internal consistency was found for the Italian version of the scale administered to a sample of non-clinical women, with a Cronbach’s alpha value of 0.85 (Nerini et al., 2018).

#### Psychobiosocial Experiences

Psychobiosocial experiences were assessed on the Psychobiosocial States Scale, Trait version (PBS-ST; Robazza et al., 2016), which was developed from the original English version of the Individualized Profiling of Psychobiosocial States (Ruiz et al., 2016) and adapted to Italian language. The PBS-ST scale consists of a 15-item list (8 functional and 7 dysfunctional) to evaluate: (a) affective, cognitive, motivational, and volitional modalities, comprising the psychological component of psychobiosocial experiences; (b) bodily and motor-behavioral modalities, which form the biological component; and (c) operational modality, included in the social component of psychobiosocial experiences, which reflects feelings of effective or ineffective performance established by social criteria expressed objectively or subjectively, such as performance scores and rankings. Each modality is measured on an item comprising three to five descriptors. For example, the item to assess the pleasant/functional affective modality is “enthusiastic, confident, carefree, joyful,” while the item to assess dysfunctional anxiety is “worried, apprehensive, concerned, troubled.” Other examples of items are “alert, focused, attentive” (functional cognitive modality), “distracted, overloaded, doubtful, confused” (dysfunctional cognitive), “purposeful, determined, persistent, decisive” (functional volitional), “unwilling, undetermined, indecisive” (dysfunctional volitional), “vigorous, energetic, physically-charged” (functional bodily), “physically-tense, jittery, tired, exhausted” (dysfunctional bodily), “effective, skillful, reliable, consistent task execution” (functional operational) and “ineffective, unskillful, unreliable, inconsistent-task execution” (dysfunctional operational). Participants were required to think about how they usually feel in their sport context, and to rate each item on a 5-point Likert scale, ranging from 0 (*not at all*) to 4 (*very, very much*). The two-factor structure of the PBS-ST was supported in a sample of Italian athletes with CFI = 0.950, TLI = 0.942, RMSA = 0.038, SRMR = 0.048 (Robazza et al., 2016). Cronbach alpha values were 0.788 (intensity of functional experiences) and 0.745 (intensity of dysfunctional experiences). Previous research has successfully applied hedonic tone and functionality distinctions to the assessment of a range of psychobiosocial experiences in physical education (Bortoli and Robazza, 2007; Bortoli et al., 2017) and sport (Robazza et al., 2016; Morano et al., 2020a), supporting the use of the scale as a valid and reliable measure in both contexts.

#### Burnout

An Italian version of the 15-item Athlete Burnout Questionnaire (ABQ; Raedeke and Smith, 2001) was used to assess participants’ burnout symptoms. This multidimensional inventory consists of three subscales, with 5 items each, measuring the following burnout dimensions: (a) emotional and physical exhaustion (e.g., “I feel overly tired from my sport participation”), (b) reduced sense of accomplishment (e.g., “I am not performing up to my ability in sport”), and (c) sport devaluation (e.g., “I’m not into sport like I used to be”). Participants rated the frequency of feeling a certain way on a 5-point Likert scale ranging from 1 (*almost never*) to 5 (*almost always*) relative to their sport experience during the current season. Evidence was provided in support of the ABQ as a reliable and valid measure of sport burnout in a population of adolescent athletes (Raedeke and Smith, 2004). In a sample of Italian adolescent athletes, the ABQ was found to have acceptable factor validity and internal consistency with Cronbach alpha values of 0.81 (emotional/physical exhaustion), 0.72 (reduced sense of accomplishment), and 0.77 (sport devaluation; Vitali et al., 2015).

### Data Analysis

Data were preliminarily examined for missing values, distribution, and potential multivariate outliers (Hair et al., 2019). Descriptive statistics (mean ± SD) and Pearson product-moment correlation coefficients (*r*) were calculated for all the studied variables and were interpreted according to Zhu’s (2012) indications—that is, 0–0.19 = no correlation, 0.20–0.39 = low correlation, 0.40–0.59 = moderate correlation, 0.60–0.79 = moderately high correlation, and > 0.80 = high correlation.

To preliminarily assess the factorial structure of the PSDQ, SPA-7, PBS-ST, and ABQ scales in the sample of the current study, we performed confirmatory factor analyses (CFA) using the maximum likelihood parameter estimates (MLM) with standard errors and a mean-adjusted chi-square test statistic, which is robust to non-normality (Byrne, 2012). Path analysis was then conducted to test the hypothesized relationships among antecedent variables (i.e., global physical self-perception, self-esteem, sport competence, body dissatisfaction, and social physique anxiety), mediators (i.e., functional and dysfunctional psychobiosocial experiences), and outcome variables (i.e., burnout modalities) in the four gender-by-age categories. To test the hypothesized mediating effects, we used the bias-corrected bootstrap method based on 5,000 resamples. Significant mediation is assumed when zero is not included in the 95% confidence intervals (MacKinnon, 2008). CFA and path analysis were conducted using Mplus 8.5 (Muthén and Muthén, 2017).

Finally, multivariate analysis of variance (MANOVA) was performed on the mean scores of the dependent variables to examine differences by gender and age categories (i.e., 12–14 vs. 15–17 years). Estimates of the effect size (partial eta squared, η_p_^2^) are provided for significant findings, with values of 0.01, 0.06, and 0.14 indicating small, medium, and large effects, respectively (Cohen, 1988).

## Results

The data screening of the whole sample showed no missing data or multivariate outliers using Mahalanobis’ distance criterion. Descriptive statistics and correlation coefficients for the data of the whole sample are reported in Table 1, while gender and age differences are shown in Table 2. CFA results on the PSDQ, SPA-7, PBS-ST, and ABQ scales provided support to the hypothesized factor structure of the measures (Table 3). Most values of comparative fit index (CFI) and Tucker Lewis fit index (TLI) were >0.90, and root mean square error of approximation (RMSEA) and standardized root mean square residual (SRMR) were <0.08, which are considered reflective of acceptable fit (Gunzler et al., 2021). Cronbach’ alpha (α) and omega (ω) reliability values were also acceptable (>0.70).

**TABLE 1:** Descriptive statistics and Pearson product-moment correlation coefficients for the whole sample (N = 640).

	Age 12–14 years		Age 15–17 years									
	Girls (*n* = 176)	Boys (*n* = 162)	Girls (*n* = 142)	Boys (*n* = 160)								
			
Variable	*M* ± *SD*	*M* ± *SD*	*M* ± *SD*	*M* ± *SD*	1	2	3	4	5	6	7	8
1. Global physical self-perception	4.73 ± 1.19	4.88 ± 1.01	4.10 ± 1.33	4.71 ± 1.05	–							
2. Self-esteem	4.84 ± 0.83	4.99 ± 0.65	4.67 ± 0.87	4.91 ± 0.72	0.53^§^	–						
3. Sport competence	7.67 ± 1.17	7.88 ± 1.28	7.46 ± 1.20	7.71 ± 1.24	0.29*	0.36*	–					
4. Social physique anxiety	2.28 ± 0.83	1.83 ± 0.61	2.51 ± 0.94	1.87 ± 0.69	–0.63^†^	–0.36*	–0.28*	–				
5. Functional experiences	2.49 ± 0.62	2.73 ± 0.56	2.48 ± 0.56	2.67 ± 0.52	0.28*	0.42^§^	0.39*	–0.19	–			
6. Dysfunctional experiences	0.47 ± 0.39	0.48 ± 0.40	0.49 ± 0.44	0.58 ± 0.45	–0.26*	–0.42^§^	–0.21*	0.23*	–0.28*	–		
7. Emotional and physical exhaustion	2.05 ± 0.76	2.23 ± 0.90	2.07 ± 0.81	2.34 ± 0.95	–0.14	–0.17	–0.02	0.11	–0.14	0.26*	–	
8. Reduced sense of accomplishment	2.15 ± 0.74	2.02 ± 0.65	2.34 ± 0.79	2.32 ± 0.75	–0.35*	–0.49^§^	–0.40^§^	0.29*	–0.45^§^	0.39*	0.24*	–
9. Sport devaluation	1.35 ± 0.59	1.39 ± 0.56	1.58 ± 0.62	1.61 ± 0.71	–0.22*	–0.29*	–0.08	0.18	–0.31*	0.31*	0.36*	0.46^§^

Correlation *low, ^§^moderate, ^†^moderately high.

**TABLE 2:** Gender and age univariate follow-up comparisons.

Independent variable	Dependent variable	*F*(1, 636)	*p*	η_p_^2^
**Gender**				
	Global physical self-perception	17.753	>0.001	0.027
	Self-esteem	10.274	0.001	0.016
	Sport competence	35.239	>0.001	0.052
	Social physique anxiety	79.874	>0.001	0.112
	Functional experiences	22.347	>0.001	0.034
	Dysfunctional experiences	2.275	0.132	0.004
	Emotional and physical exhaustion	11.046	0.001	0.017
	Reduced sense of accomplishment	1.554	0.213	0.002
	Sport devaluation	0.501	0.479	0.001
**Age**				
	Global physical self-perception	19.127	>0.001	0.029
	Self-esteem	4.363	0.037	0.007
	Sport competence	0.258	0.612	0.000
	Social physique anxiety	4.475	0.035	0.007
	Functional experiences	0.666	0.415	0.001
	Dysfunctional experiences	2.694	0.101	0.004
	Emotional and physical exhaustion	0.928	0.336	0.001
	Reduced sense of accomplishment	18.032	>0.001	0.028
	Sport devaluation	21.049	>0.001	0.032

**TABLE 3:** Confirmatory Factor Analysis Fit Indices and Reliability Values of the Measures.

Instrument	Factor (number of items)	χ^2^/*df*	CFI	TLI	RMSEA (90% CI)	SRMR	α	ω
PSDQ		4.523	0.947	0.934	0.074 (0.065–0.084)	0.045		
	Global physical self-perception (6)						0.943	0.946
	Self-esteem (6)						0.737	0.727
SPA-7		5.370	0.949	0.924	0.083 (0.065–0.102)	0.036		
	Social physique anxiety (7)						0.829	0.828
PBS-ST		2.681	0.909	0.893	0.051 (0.044–0.059)	0.051		
	Functional (8)						0.832	0.834
	Dysfunctional (7)						0.733	0.738
ABQ		3.086	0.913	0.895	0.057 (0.049–0.065)	0.060		
	Emotional and physical exhaustion (5)						0.815	0.818
	Reduced sense of accomplishment (5)						0.763	0.764
	Sport devaluation (5)						0.747	0.782

*PSDQ, Physical Self-Description Questionnaire; SPA-7, Social Physique Anxiety; PBS-ST, Psychobiosocial States Scale, Trait version; ABQ, Athlete Burnout Questionnaire; χ^2^/df, chi-square/degrees of freedom; CFI, comparative fit index; TLI, Tucker Lewis fit index; RMSEA, root mean square error of approximation; SRMR, standardized root mean square residual; α, Cronbach’s alpha; ω, omega.*

Correlation analysis (Table 1) indicated that both global physical self-perception and self-esteem scores were positively related to sport competence and functional experiences, and negatively related to dysfunctional experiences, social physique anxiety, and all burnout subscale scores. Moreover, functional experiences correlated positively with sport competence and negatively with burnout subscales, whereas dysfunctional experiences correlated positively with social physique and burnout subscales. All correlations were in the expected direction, with magnitude of coefficients ranging from low to moderately high (Zhu, 2012).

**FIGURE 1:**
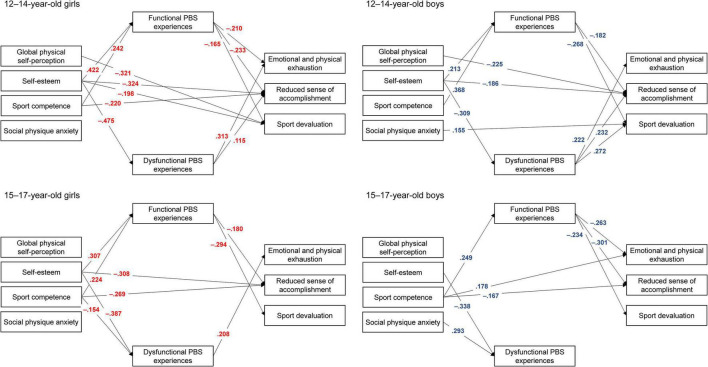
Interrelationships between self-perceptions, psychobiosocial experiences and burnout across age and gender. Only significant standardized estimates are presented (*p* < 0.05).

Path analysis (Figure 1) showed significant direct effects in the expected direction from self-esteem and/or sport competence to reduced sense of accomplishment across gender and age categories. In 12–14-year-old girls, negative direct effects were observed from global physical self-perception and self-esteem to sport devaluation. In 12–14-year-old boys, negative direct effects emerged from global physical self-perception to reduced sense of accomplishment, and positive direct effects from social physique anxiety to sport devaluation. These findings suggest that higher scores on global physical self-perception, self-esteem, and sport competence were related to lower burnout symptoms, while higher scores on social physique anxiety were linked to higher scores on sport devaluation. Contrary to our hypotheses, a significant positive link was observed from sport competence to emotional and physical exhaustion in 15–17-year-old boys.

Mediation analysis across gender and age categories revealed significant indirect effects. Specifically, the results indicated significant indirect effects of self-esteem on all three burnout dimensions (i.e., emotional and physical exhaustion, reduced sense of accomplishment, and sport devaluation) via both functional and dysfunctional psychobiosocial experiences (see Supplementary Table 1). The results also indicated significant indirect effects of sport competence on all three burnout dimensions via functional experiences. Significant indirect effects of sport competence on emotional and physical exhaustion via dysfunctional experiences emerged in the case of 15–17–year–old girls. All indirect effects were in the expected direction.

Multivariate analysis of variance yielded significant results by gender, Wilk’s λ = 0.789, *F*(9, 628) = 18.642, *p* < 0.001, η_p_^2^ = 0.211, and by age category, Wilk’s λ = 0.929, *F*(9, 629) = 4.950, *p* < 0.001, η_p_^2^ = 0.071, while gender by age interaction was not significant (*p* = 0.104). Univariate follow-up showed significant differences by gender and age in several studied variables (see mean scores in Table 1, and gender and age comparisons in Table 2). Compared to girls, boys reported higher mean scores on competence, functional psychobiosocial experiences, global physical self-perception, self-esteem, emotional and physical exhaustion, and lower mean scores on social physique anxiety. Moreover, compared to 12–14-year-olds, 15–17-year-olds reported lower scores on global physical self-perception and self-esteem, and higher scores on social physique anxiety, reduced sense of accomplishment, and sport devaluation.

## Discussion

This study examined the interplay between self-perceptions (i.e., global physical self-perception, self-esteem, sport competence, social physique anxiety) and psychobiosocial experiences in predicting burnout symptoms in early and middle adolescent athletes. The study contributes to the literature on burnout in sport by investigating intrapersonal factors which have received scarce research attention. The high mean scores on global physical self-perception, self-esteem, sport competence, and functional psychobiosocial experiences, and the low mean scores on social physique anxiety, dysfunctional psychobiosocial experiences, and burnout symptoms reported by the participants suggest sport participation to be in general a positive experience for adolescents.

As expected, global physical self-perception, self-esteem, and sport competence were positively interrelated and negatively related to social physique anxiety and burnout symptoms, while social physique anxiety was positively related to burnout symptoms (Table 1). The significant positive correlation values among global physical self-perception, self-esteem, and sport competence underline the crucial role of the body in the global evaluative feelings about themselves in adolescents. Notably, while in previous studies social physique anxiety was particularly considered in relation to self-esteem (e.g., Brunet et al., 2010), in line with Brunet et al.’s study findings, in our data we found a moderately high negative correlation between social physique anxiety and global physical self-perception. In the sport context, specific bodily and physical self-perception constructs seem more relevant compared to a more general self-evaluative construct.

Functional psychobiosocial experiences were positively related to global physical self-perception, self-esteem, and sport competence, and negatively related to social physical anxiety and burnout symptoms. Conversely, and in line with our hypotheses, dysfunctional psychobiosocial experiences were associated negatively with global physical self-perception, self-esteem, and sport-specific competence, and positively with social physique anxiety and burnout symptoms. These results suggest that adolescents involved in sport may experience a range of pleasant states, but also unpleasant states deriving from intrapersonal, interpersonal, and situational factors. Findings align with Weiss et al.’s (2012) contention that self-perceptions strongly predict cognitive, affective, and behavioral outcomes, such as positive and negative emotions, motivational orientations, performance, and health-related behaviors.

### Path Analysis and Indirect Effects

Overall, and in line with our hypothesis, path analysis results showed significant direct links between self-perceptions (i.e., global physical self-perception, self-esteem, and sport competence) and burnout symptoms. Different patterns of relationships were observed across gender and age. Significant positive links emerged between self-esteem and reduced sense of accomplishment in girls and younger boys (Figure 1). Direct links were also observed between sport competence and reduced sense of accomplishment for girls and older boys. Global physical self-perception was negatively associated with sport devaluation in younger girls and with reduced sense of accomplishment in boys. Noteworthy, social physique anxiety (i.e., negative feelings about the body) was positively related with sport devaluation in younger boys. Our findings suggest that positive self-perceptions represent possible protective factors toward burnout.

As expected, significant negative links were found between functional experiences and burnout dimensions, whereas positive links were observed between dysfunctional experiences and burnout, especially for younger participants. Indirect effects emerged between self-esteem and burnout via functional and dysfunctional psychobiosocial experiences. Significant indirect links emerged between sport competence and reduced sense of accomplishment, and sport devaluation via functional psychobiosocial experiences.

Self-esteem is viewed as a general construct reflecting a global evaluative meaning about themselves, whereas sport competence is regarded as the perceptions about the level of specific physical and technical sport skills during training and competition (Marsh et al., 1994). Therefore, both the general evaluative dimension of the self and sport specific competence appear to be decisive in giving value and meaning to the sport experience. This contention is in line with a study with early adolescents in which perceived sports competence was strongly linked to global self-esteem (Rubeli et al., 2020). The development of sport specific abilities and skills is therefore associated with positive self-esteem in young athletes, as well as a range of pleasant and functional emotions and emotion-related states (Bortoli et al., 2011).

In explaining the patterns of relationships across gender and age, one could consider that adolescent growth spurts begin at different times in girls and boys, 10 years of age for girls and around 12 years of age for boys; puberty begins earlier for girls, around age 11 or 12, and for boys around age 13 or 14 (Feldman, 2017). In addition, there are wide developmental variations among individuals, including physical, emotional, cognitive, and social changes, which can adversely affect sport participation and performance (Gustafsson et al., 2011; Harter, 2012; Gerber et al., 2019). For 12–14-year-olds, the sport structure is already formalized and competitive, with performance and victory being often emphasized at this age level. In Italy, for instance, participants under 12 years of age are already involved in national championships in some sports. Individual differences in pubertal development influence performances, especially in younger boys. Also due to cultural gender-related stereotypes, boys, especially those who mature later, may become concerned with their physical and athletic prowess; in this case, boys may diminish the value they attribute to sport in an attempt to better manage their sport experience.

Contrary to our hypotheses, a significant positive link was observed from sport competence to emotional and physical exhaustion in 15–17-year-old boys. Given that physical/emotional exhaustion is associated with intense training and competition (Martin and Horn, 2013), this result may depend on the greater competitive commitment and the growing pressure placed on young athletes by coaches, parents, and the sport environment in general. Thus, it may be that as these boys grow up and their sport competence increases, so does the pressure and expectations that lead to higher levels of exhaustion (Gustafsson, 2007; Martin and Horn, 2013). Further research to ascertain the possible determining factors is needed.

The present study also highlights the role of psychobiosocial experiences on burnout symptoms. The results indicated significant indirect effects of self-esteem and sport competence on the burnout dimensions for girls and younger boys via both functional and dysfunctional psychobiosocial experiences, while sport competence showed indirect effects on the burnout dimensions for older boys through functional psychobiosocial experiences. Our results suggest that positive self-perceptions foster functional experiences that in turn prevent from burnout symptoms, also by diminishing dysfunctional experiences that are linked to burnout symptoms. Findings concur with those of Morano et al.’s (2020a) study in which functional psychobiosocial experiences of adolescent athletes were negatively related to burnout symptoms, while dysfunctional experiences were positively related.

### Gender and Age Differences

Significant differences were found by gender, with boys reporting higher scores on competence, global physical self-perception, self-esteem than girls, and lower scores on social physique anxiety. For adolescents, the sport context represents an important life domain that provides opportunities to develop physical skills and social relationships and to obtain relevant health benefits. However, the effects of sport participation on self-perceptions differ by gender. For example, Balaguer et al. (2012) found that sport participation was positively associated with athletic competence for both boys and girls, but for boys it was also associated with physical appearance and social acceptance. In a study investigating perceptions about constraints to sport participation in middle school students (Casper et al., 2011), girls reported significantly more limiting factors toward continued sport participation than boys, lower confidence and self-esteem in physical and sport activities, less social approval, as well as more restrictions to sport participation because of higher household tasks and family responsibilities imposed on them. In another study in a high-performance youth sport environment, boys reported higher perceptions of confidence and self-competence, and a stronger connection with their coach compared to girls (O’Connor et al., 2020). To explain gender differences, research in social psychology has emphasized the role of cultural stereotypes learned and integrated in the self during the socialization process, underlining that sport is generally considered a masculine domain (Fredricks and Eccles, 2005). In a large sample of 11–18-year-olds, Hagger and Stevenson (2010) reported girls to experience higher levels of social physique anxiety. Different gender socialization modalities involve the importance attributed to physical appearance, which results in higher levels of social physique anxiety in women across the lifespan (for a review in sport, see Sabiston et al., 2014).

Gender differences were also found in psychobiosocial experiences, with boys showing higher intensities of functional experiences than girls. For boys, participation in sport can be more central in their lives than girls. They are often involved in more than one sport, invest much of their time, and attain great enjoyment (Ricciardelli et al., 2006) and more pleasant emotional experiences (Morano et al., 2020a). However, in the current study boys also reported higher burnout symptoms in terms of emotional and physical exhaustion. This finding on male athletes could reflect higher personal and social performance demands deriving, for example, from parents who play a key role in transmitting gender stereotypes. Indeed, parents of boys have been shown to consider sport as more important compared to parents of girls (Fredricks and Eccles, 2005). In boys, therefore, it could be hypothesized that a greater investment in sport participation has both a bright and a dark side: the first reflected in a range of emotion-related functional experiences, and the second deriving from higher personal and social pressures toward performance leading to burnout symptoms.

Significant differences also emerged by age, with 15–17-year-olds reporting lower scores on global physical self-perception and self-esteem compared to 12–14-year-olds, and higher scores on social physique anxiety, reduced sense of accomplishment, and sport devaluation. A decline in self-esteem from early to middle adolescence was observed by Marsh (1989) and attributed to the uncertainties linked to physical and social changes during this period. Adolescence represents a crucial developmental transition, with physical changes, cognitive-developmental advances, and enhanced social expectations (Salmela-Aro, 2011). In early adolescence, the self becomes increasingly differentiated, with a proliferation of self-representations, highly compartmentalized, distinct from each other, which vary as a function of the social context. A high concern regarding others’ perceptions of the self can contribute to the need to protect and enhance the self-value and determine unrealistic highly positive self-representations. In middle adolescence, instead, self-awareness and the ability to make comparisons between single abstractions about self-dimensions increase, and awareness quickly shifts among images of self that are defined quite differently. Adolescents become extremely preoccupied with opinions and expectations of significant others, which represent a source of doubt and confusion. The resulting global self-perception and self-esteem decline (Harter, 2012) also involves perceptions of physical appearance. This can explain the higher level of social physique anxiety in older female and male participants.

The higher scores on reduced sense of accomplishment and sport devaluation observed in middle adolescent athletes are in line with previous findings (Harris and Watson, 2014; Isoard-Gautheur et al., 2015). As middle adolescents grow, performance pressure may also increase, with subsequent greater demands for time and commitment required for training and competitions. At the same time, a challenge for middle adolescent athletes is to integrate the demands of their athletic lives with other important developmental opportunities, including school and friendship. Moreover, in this development phase adolescents expand the ability to use more external feedback sources to conceptualize their sense of sport achievement, with a more realistic evaluation of their own sport skills that oftentimes can result in a reduced sense of accomplishment (Harris and Watson, 2014). Middle adolescents also reported higher scores in sport devaluation, reflecting a negative and detached attitude toward sport and the involvement in it, which results in a lack of concern and care for sport and performance quality (Raedeke, 1997). This result may be due to adolescents’ use of a self-protective mechanism, a process called discounting, which minimizes the importance of aspects of life that elicit feelings of inadequacy or lack of competence (Harter, 2012). When perceived competence in sport tasks is low, or an imbalance between sport demands and personal abilities is perceived, young athletes can still maintain high self-esteem if they discount the value of the activities they are involved in.

## Conclusion

This study extends existing literature on the antecedents of burnout in sport by examining the interplay between self-perceptions, burnout symptoms, and psychobiosocial experiences in adolescent athletes. The findings reported here shed light on intrapersonal factors—namely, global physical self-perception, self-esteem, sport competence, and social anxiety—which have received little research attention. The study also extends the current knowledge of the relationships between emotions and burnout shifting from anxiety (e.g., Gomes et al., 2017) and other selected emotions (e.g., González-García et al., 2020) to a broader range of emotion-related experiences associated with sport participation. The results of this investigation suggest that enhanced global physical self-perception, self-esteem, and sport competence could counteract burnout symptoms, and that functional experiences can play a positive role in the relationship between these factors and burnout. Self-esteem, or global self-worth, is related to self-perceptions of competence originating from experiences occurring in diverse personal, environmental, and social contexts. Sport provides opportunities for adolescents to improve psychological skills such as self-discipline, leadership, self-awareness, and self-confidence, which foster the development of positive self-esteem and self-concept (Ricciardelli and Yager, 2016).

The present study also provided evidence of the role of psychobiosocial experiences on burnout symptoms. Functional experiences (e.g., feeling joyful, focused, energetic, and effective) were positively related to positive self-perceptions and negatively to burnout symptoms, while dysfunctional experiences (e.g., feeling worried, doubtful, tired, and unskillful) showed opposite relationships. Therefore, psychobiosocial experiences seem to play an important role not only on sport participation, but also on burnout prevention. Functional and pleasant experiences can lead to more empowering, enriched, and positive sport participation. Functional experiences and enhanced physical self-perception, self-esteem, and sport competence can be pursued through the implementation of a variety of coaching strategies. For example, drawing on several theoretical and empirically tested coaching frameworks (see Resende and Gomes, 2020), Bloom et al. (2020) suggested coaching strategies to build learning environments that meet youth athletes’ needs and facilitate the development of positive outcomes. Some of these strategies involve focusing on learning and personal development over winning (e.g., promoting and praising effort, enjoyment, and persistence, avoiding punishment or punitive behaviors particularly after mistakes, communicating a belief in athletes’ ability to improve), creating a supportive environment (e.g., setting realistic expectations, focusing on athletes’ strengths, talents, and interests), and providing all athletes with opportunities for leadership, decision making, and problem-solving.

We acknowledge that the cross-sectional nature of this study is a limitation which makes it difficult to draw conclusions about the direction of the relationships or causality. Therefore, future research should incorporate the practical suggestions indicated above in longitudinal or intervention studies to gain a deeper understanding of the interplay among environmental factors, intrapersonal factors, psychobiosocial experiences, and burnout symptoms. Another limitation is the use of a single-item scale to assess perceived sport competence. Although single-item scales possess high face validity in evaluating global constructs, future research including the use of multi-item scales capturing different facets of these constructs is warranted. Furthermore, self-report measures should be complemented with objective assessments of pubertal maturation and morphological indices (e.g., BMI), in order to take account of the physical changes associated with the onset and progression of puberty across different stages of development.

## Data Availability Statement

The raw data supporting the conclusions of this article will be made available by the authors, without undue reservation.

## Ethics Statement

The studies involving human participants were reviewed and approved by Ethics Committee of the University of Chieti-Pescara (Italy). Written informed consent to participate in this study was provided by the participants’ legal guardian/next of kin.

## Author Contributions

MM, CR, MR, and LB: conceptualization, methodology, writing—original draft, and writing—review and editing. CR and LB: data management and data curation. CR and MR: formal analysis. All authors contributed to the article and approved the submitted version.

## Conflict of Interest

The authors declare that the research was conducted in the absence of any commercial or financial relationships that could be construed as a potential conflict of interest.

## Publisher’s Note

All claims expressed in this article are solely those of the authors and do not necessarily represent those of their affiliated organizations, or those of the publisher, the editors and the reviewers. Any product that may be evaluated in this article, or claim that may be made by its manufacturer, is not guaranteed or endorsed by the publisher.
