# Supplementary material for: Sport Participation in Early and Middle Adolescence: The Interplay Between Self-Perception and Psychobiosocial Experiences in Predicting Burnout Symptoms
Source: Front Psychol. 2022 Jun 13;13:855179. doi: 10.3389/fpsyg.2022.855179 (PMC9235354; doi:10.3389/fpsyg.2022.855179)
Supplement: Supplementary file 1 [file Table_1.pdf]

Supplementary Table 1. Total, total indirect, indirect, and direct effects for paths from antecedent variables to burnout variables via functional psychobiosocial experiences (FE) and dysfunctional psychobiosocial experiences (DE).

Effect	Standardized estimate (β) and bootstrap bias–corrected 95% confidence interval			
	12–14–year–old girls	12–14–year–old boys	15–17–year–old girls	15–17–year–old boys
Global physical self-perception (GPSP) to Emotional and physical exhaustion (EPE)				
Total	–.081 (–.303, .167)	–.057 (–.221, .118)	–.143 (–.390, .083)	.125 (–.146, .392)
Total indirect	.000 (–.094, .093)	–.002 (–.056, .044)	–.003 (–.053, .046)	–.047 (–.144, .019)
GPSP → FE → EPE	–.013 (–.078, .027)	.000 (–.024, .016)	.000 (–.035, .027)	–.047 (–.141, .006)
GPSP → DE → EPE	.013 (–.051, .097)	–.002 (–.055, .040)	–.002 (–.049, .033)	.001 (–.025, .048)
GPSP → EPE	–.082 (–.307, .175)	–.054 (–.217, .114)	–.140 (–.389, .071)	.172 (–.097, .443)
Global physical self-perception (GPSP) to Reduced sense of accomplishment (RSA)				
Total	–.009 (–.178, .177)	**–.228 (–.379, –.080)**	.163 (–.025, .345)	.003 (–.251, .250)
Total indirect	–.009 (–.076, .049)	–.003 (–.060, .053)	.000 (–.050, .047)	–.041 (–.148, .061)
GPSP → FE → RSA	–.014 (–.076, .029)	–.001 (–.038, .030)	.001 (–.040, .049)	–.054 (–.158, .011)
GPSP → DE → RSA	.005 (–.018, .040)	–.002 (–.049, .044)	–.001 (–.030, .016)	.013 (–.021, .090)
GPSP → RSA	.001 (–.176, .190)	**–.225 (–.376, –.077)**	.163 (–.026, .339)	.044 (–.226, .311)
Global physical self-perception (GPSP) to Sport devaluation (SD)				
Total	**–.323 (–.586, –.045)**	.023 (–.150, .204)	.090 (–.171, .328)	.088 (–.141, .311)
Total indirect	–.002 (–.075, .071)	–.004 (–.076, .061)	.000 (–.076, .072)	–.032 (–.131, .046)
GPSP → FE → SD	–.010 (–.064, .020)	–.002 (–.048, .040)	.002 (–.060, .078)	–.042 (–.141, .007)
GPSP → DE → SD	.008 (–.022, .096)	–.002 (–.058, .048)	–.002 (–.033, .027)	.010 (–.012, .085)
GPSP → SD	**–.321 (–.585, –.057)**	.027 (–.143, .189)	.090 (–.153, .317)	.120 (–.107, .369)
Self-esteem (SE) to Emotional and physical exhaustion (EPE)				
Total	**–.286 (–.473, –.080)**	–.049 (–.256, .140)	–.017 (–.243, .189)	**–.257 (–.451, –.050)**
Total indirect	**–.237 (–.381, –.127)**	**–.080 (–.188, –.014)**	–.054 (–.161, .043)	–.038 (–.133, .041)
SE → FE → EPE	**–.089 (–.185, –.017)**	–.011 (–.067, .032)	.026 (–.035, .101)	–.036 (–.114, .010)
SE → DE → EPE	**–.149 (–.271, –.066)**	**–.068 (–.167, –.016)**	**–.080 (–.181, –.015)**	–.002 (–.081, .066)
SE → EPE	–.049 (–.252, .161)	.031 (–.154, .194)	.038 (–.182, .244)	–.218 (–.431, .008)
Self-esteem (SE) to Reduced sense of accomplishment (RSA)				
Total	**–.477 (–.615, –.345)**	**–.297 (–.430, –.161)**	**–.401 (–.574, –.221)**	–.188 (–.382, .011)
Total indirect	**–.153 (–.267, –.072)**	**–.110 (–.193, –.054)**	**–.093 (–.188, –.014)**	**–.095 (–.203, –.013)**
SE → FE → RSA	**–.098 (–.191, –.035)**	**–.039 (–.101, –.004)**	**–.055 (–.122, –.015)**	–.041 (–.118, .012)
SE → DE → RSA	**–.054 (–.119, –.008)**	**–.072 (–.145, –.026)**	–.037 (–.116, .018)	**–.054 (–.144, –.002)**
SE → RSA	**–.324 (–.465, –.181)**	**–.186 (–.322, –.055)**	**–.308 (–.502, –.117)**	–.093 (–.267, .092)
Self-esteem (SE) to Sport devaluation (SD)				
Total	**–.364 (–.535, –.180)**	**–.239 (–.421, –.037)**	–.177 (–.346, .017)	**–.258 (–.438, –.052)**
Total indirect	**–.165 (–.305, –.054)**	**–.141 (–.250, –.064)**	**–.147 (–.262, –.064)**	**–.072 (–.161, –.002)**
SE → FE → SD	**–.069 (–.158, –.005)**	**–.057 (–.122, –.015)**	**–.090 (–.185, –.030)**	–.032 (–.116, .008)
SE → DE → SD	–.096 (–.243, .000)	**–.084 (–.182, –.022)**	–.057 (–.157, .004)	–.040 (–.134, .015)
SE → SD	**–.198 (–.382, –.030)**	–.098 (–.264, .078)	–.030 (–.216, .179)	–.186 (–.386, .026)
Sport competence (SC) to Emotional and physical exhaustion (EPE)				
Total	.005 (–.156, .195)	.034 (–.142, .203)	.051 (–.088, .188)	.112 (–.033, .269)
Total indirect	–.022 (–.106, .060)	–.041 (–.133, .034)	–.013 (–.077, .047)	**–.066 (–.144, –.020)**
SC → FE → EPE	**–.051 (–.121, –.010)**	–.020 (–.106, .050)	.019 (–.022, .081)	**–.065 (–.147, –.019)**
SC → DE → EPE	.029 (–.021, .097)	–.021 (–.088, .012)	**–.032 (–.085, –.004)**	–.001 (–.035, .027)
SC → EPE	.027 (–.128, .196)	.075 (–.133, .252)	.064 (–.083, .209)	**.178 (.039, .327)**
Sport competence (SC) to Reduced sense of accomplishment (RSA)				
Total	**–.266 (–.405, –.096)**	–.120 (–.239, .009)	**–.324 (–.494, –.134)**	**–.263 (–.397, –.103)**
Total indirect	**–.046 (–.108, –.001)**	**–.089 (–.181, –.014)**	**–.055 (–.119, –.011)**	**–.095 (–.189, –.030)**
SC → FE → RSA	**–.057 (–.119, –.019)**	**–.067 (–.151, –.007)**	**–.040 (–.095, –.009)**	**–.075 (–.163, –.022)**
SC → DE → RSA	.011 (–.005, .046)	–.022 (–.077, .018)	–.015 (–.058, .006)	–.021 (–.070, .001)
SC → RSA	**–.220 (–.365, –.061)**	–.031 (–.164, .122)	**–.269 (–.442, –.087)**	**–.167 (–.296, –.021)**
Sport competence (SC) to Sport devaluation (SD)				
Total	.055 (–.112, .211)	.015 (–.148, .180)	.016 (–.144, .165)	.088 (–.088, .296)
Total indirect	–.021 (–.087, .054)	**–.124 (–.217, –.048)**	**–.089 (–.180, –.028)**	**–.073 (–.162, –.022)**
SC → FE → SD	**–.040 (–.102, –.005)**	**–.098 (–.178, –.040)**	**–.066 (–.155, –.016)**	**–.058 (–.138, –.014)**
SC → DE → SD	.019 (–.006, .089)	–.026 (–.099, .018)	–.023 (–.070, .002)	–.015 (–.073, .004)
SC → SD	.076 (–.090, .242)	.139 (–.015, .311)	.104 (–.053, .254)	.161 (–.017, .366)

Supplementary Table 1 continues

Supplementary Table 1 continued

Social physique anxiety (SPA) to Emotional and physical exhaustion (EPE)				
Total	.002 (–.193, .223)	.076 (–.078, .240)	.059 (–.155, .288)	**.230 (.011, .440)**
Total indirect	.016 (–.057, .099)	.014 (–.016, .065)	.030 (–.013, .115)	.006 (–.082, .097)
SPA → FE → EPE	–.011 (–.063, .026)	–.002 (–.032, .011)	.004 (–.011, .062)	.004 (–.056, .085)
SPA → DE → EPE	.027 (–.022, .099)	.015 (–.008, .065)	.026 (–.006, .099)	.002 (–.054, .081)
SPA → EPE	–.014 (–.208, .196)	.063 (–.089, .223)	.029 (–.175, .238)	.224 (–.014, .455)
Social physique anxiety (SPA) to Reduced sense of accomplishment (RSA)				
Total	.106 (–.053, .279)	.060 (–.093, .227)	.152 (–.029, .323)	**.229 (.029, .425)**
Total indirect	–.002 (–.051, .056)	.011 (–.033, .062)	.004 (–.038, .054)	.052 (–.049, .170)
SPA → FE → RSA	–.012 (–.061, .030)	–.005 (–.045, .022)	–.008 (–.055, .028)	.005 (–.065, .085)
SPA → DE → RSA	.010 (–.006, .044)	.016 (–.010, .063)	.012 (–.006, .061)	.047 (.000, .136)
SPA → RSA	.108 (–.045, .270)	.049 (–.110, .209)	.148 (–.028, .318)	.178 (–.021, .398)
Social physique anxiety (SPA) to Sport devaluation (SD)				
Total	–.119 (–.317, .091)	**.166 (.021, .334)**	.073 (–.147, .264)	**.256 (.043, .469)**
Total indirect	.009 (–.043, .087)	.011 (–.044, .079)	.006 (–.070, .073)	.038 (–.056, .152)
SPA → FE → SD	–.009 (–.057, .019)	–.008 (–.054, .033)	–.013 (–.083, .048)	.004 (–.059, .072)
SPA → DE → SD	.017 (–.009, .097)	.019 (–.011, .075)	.019 (–.005, .087)	.035 (–.013, .140)
SPA → SD	–.127 (–.319, .069)	**.155 (.024, .304)**	.067 (–.134, .265)	.217 (–.006, .439)

Note. In bold, significance indicated via 95% confidence interval.
